# Supplementary material for: Endotracheal tube, by the venturi effect, reduces the efficacy of increasing inlet pressure in improving pendelluft
Source: PLoS One. 2023 Sep 14;18(9):e0291319. doi: 10.1371/journal.pone.0291319 (PMC10501657; doi:10.1371/journal.pone.0291319)
Supplement: Attachment — Submitted filename: Response to Reviewers.docx [file pone.0291319.s001.docx]

Response to Review Comments

Please use the space provided to explain your answers to the questions above. You may also include additional comments for the author, including concerns about dual publication, research ethics, or publication ethics. (Please upload your review as an attachment if it exceeds 20,000 characters)

Reviewer #1:

**Comment 1**: This bench research aimed to investigate via computer simulation whether endotracheal tube and ventilatory support contribute to pendelluft. Honestly, this paper is very hard to read and some sections are misleading.
I don't feel it suitable for publication in this journal.

**Response:** We have taken the reviewer's opinion seriously and have made significant changes to the manuscript.

Particularly in the Introduction section, we explain that pendelluft is a concept related to P-SILI, and that this concept has been gaining attention in recent years. We also explain that the exacerbating factors of pendelluft are not fully understood; we therefore investigated some of them (ventilatory support and ETT) using computer simulations.

Furthermore, we used a professional English editing service to improve the readability of the manuscript.

Reviewer #2:

**Comment 1**. *The reference to figure 1 has already been described in materials and methods (simulation model). So, there is no need to introduce it again in results (sentence 176).*

**Response**: Thank you for your comment. Per your recommendation, I have deleted the sentence.

**Comment 2.** *Line 208. Sentence must be corrected: "and the gas flow into the models was not observed at the other outlets."*

**Response:** Thank you for your useful advice. The sentence was misleading. Therefore, we have defined pendelluft in the Materials and Methods section as follows:

“Under the boundary conditions, the gas flow out of the model through the outlets meant lung lobe inflation, whereas the gas flow into the model through the outlets meant lung lobe deflation. Therefore, in this study, gas flow into the model through the outlets was defined as the pendelluft” (Page 8 line 162 to 165 of the revised text). We have also changed the corresponding sentence in the Results section, as follows; “and the pendelluft was observed at the other outlets.” (Page 13 line 248 to 249 of the revised text)

**Comment 3**. *Figure 4: Even in the group with no effort, the outflow was greater in the LL and RL segments.
With the individual in the vertical position (standing or sitting), the base of the lung receives a greater volume of air than the apex. This model has been built in an upright position. How could the results be translated to a mechanically ventilated patient in the supine position? Put up for discussion.*

**Response:** Thank you for your useful suggestion. As you suggested, a previous study using CT scan (reference number 41) reported that the proportional volumes of bilateral upper lobes relative to the total lung volume were significantly lower in the vertical than in the supine position, whereas the proportional volumes of the bilateral lower lobes were significantly higher in the vertical than in the supine position in healthy volunteers. This study also indicated that volume change from expiration to inspiration was greater in the lower lobe than in the upper lobe. This is consistent with the result of our study that the gas flow was greater in the lower lobes than in the upper lobes.

On the other hand, in mechanically ventilated patients with ARDS, a previous study reported (reference number 66) that changing the body position from supine to seated increased end-expiratory lung volume. Furthermore, a previous study (reference number 1) described that reduced FRC causes stronger diaphragm contraction and greater negative pressure. Summarizing these reports, we could say that reduced FRC in the supine position causes stronger diaphragm contraction and greater negative pressure; this leads to a greater inter-lobar pressure difference if the negative pressure is localized in lower lobes because of this solid like behavior. Therefore, we should apply greater negative pressure (boundary condition) to the lower lobes in spine simulation than in upright simulation.

So, we added some sentences and a useful reference in the discussion section as follows: “Third, more flow was directed toward the lower lobe, even under non-effort or fluid-like effort conditions. This result is consistent with a previous report showing that volume change from expiration to inspiration was greater in the lower lobe than in the upper lobe in healthy volunteers [42].

(Page 19 line 371 to 374 of the revised text)

Also, we added some sentences and a useful reference in the limitation section as follows; “Fourth, this study did not take the patients’ position into account. Previous studies have reported that FRC in the supine position is smaller than that in the upright position; a smaller FRC is relevant to stronger diaphragm contraction, leading to more inter-lobar pressure difference caused by the solid-like behavior [1,67]. Therefore, if we investigate the change in gas flow distribution due to the body position, it may be appropriate to apply more negative pressure to the lower lobes in the supine position than in the upright position.”

(Page 23 line 458 to 464 of the revised text)

**Comment 4**. *When the airway resistance is increased the flow is totally turbulent, in this case there will be gas inertia and the linear velocity of the gas will be reduced. One concept that has emerged is the consideration of the amount of energy transferred from the ventilator to the respiratory system per unit time, which can be quantified as mechanical power (MP). Higher MP has been associated with worse clinical outcomes. It would be very interesting if the author could implement a MP calculation for the presented models. Put up for discussion.*

**Response**: We agree with the reviewer’s useful comments that mechanical power is related to the lung damage. As previously reported, inspiratory flow rate is related to the resistive component of the mechanical power. During the inspiratory phase in PCV, driving pressure generated by the ventilator is consumed in ETT and patient airway, and the rest was used to inflate the lung. Hence, immediately after inhalation begins, most of the energy from the ventilator is used for the resistive component, whereas at the end of inhalation, the percentage of resistive component decreases and more energy is used to maintain lung inflation, which is the elastic component. Therefore, recognizing the resistive component is helpful because inadequate support leads to lung injury. In this study, the pressure difference between the inlet and outlet is associated with the resistive component. Therefore, we have added the following sentences in the Discussion section:

“Additionally, we should discuss how this study is applied to the mechanical power (MP) and WOB. MP is an estimate of the mechanical energy per minute that is applied to the respiratory system. MP is divided into resistive, elastic, and PEEP components. In patients with ARDS, higher MP has been reported to be associated with higher mortality [56]. However, the individual impact of each component requires further discussion. For instance, some reports indicate that the resistive component may not be as damaging to the lung as initially expected [57,58], elastic components above a certain value have been found to be particularly harmful [59], and the PEEP component has shown protective effects on the lungs under certain conditions [60]. In this model, the resistive component is expressed as the pressure difference between the inlet and outlet pressure, and elastic component is expressed as the outlet boundary conditions. Therefore, as inspiration proceeds and the pressure difference decreases (corresponding to the outlet boundary condition change from strong to weak), the inspiratory flow will decrease and less energy will be consumed as the resistive component. At the end of the inspiration, most of the energy is used to keep the lung inflated (elastic component).”

(Page 21 line 421 to 434 of the revised text)

Reviewer #3:

**Comment 1***. Introduction
1) This model simulates regional biomechanical conditions that would occur in the context of increased respiratory effort in severe ARDS, at different frictional resistive components, with and without invasive MV support, apparently in separatesupine position.
However, it is based only on works studying respiratory effort in subjects with ARDS under MV. There are some studies in recent years that have focused on both biomechanical phenomena (regional strain, lung heterogeneity, strain progression) and their regional biological transduction (lung damage progression) in murine and porcine models of P-SILI. It is relevant information to consider within the theoretical framework.*

**Response:** We agree with the reviewer’s comment. Spontaneous breathing causes lung injury with and without mechanical ventilation, through the mechanism called P-SILI. Transpulmonary pressure increases with strong spontaneous breathing (negative pleural pressure) and lung injury progresses. In addition, in the injured lung, negative pleural pressure is unevenly distributed throughout the thoracic cavity, resulting in air movement within the lung at the onset of inspiration (pendelluft phenomenon), causing hyperextension and severe inflammation in the dorsal lung region.

According to the useful suggestion, we introduced the concept of P-SILI in the Introduction section, and have added the relevant references:

” Spontaneous breathing has long been considered desirable during mechanical ventilation because it maintains contraction of the dorsal diaphragm, resulting in improved gas exchange and air content distribution. However, recent studies have demonstrated that strong inspiratory effort aggravates lung injury in the aforementioned patients through a mechanism called patient self-inflicted lung injury (P-SILI) [1–3].”

(Page 3 line 47 to 51 of the revised text)

We also added the following text:

“Gas movement in a patients’ lung, called pendelluft, is considered one of the mechanisms of P-SILI [4].”

(Page 3 line 52 to 53 of the revised text)

We have added the following parts to the discussion section to explain our study in the framework of the P-SILI:

“Recently, pendelluft has been considered one of the mechanisms of P-SILI, a concept wherein patients’ inspiratory effort causes regional edema, tidal recruitment, and distortion [1,2,35,36]. The inhomogeneous gas distribution of pendelluft is assumed to increase the strain and amplify the stress at the interface between open and closed pulmonary units (= stress raiser), thereby damaging the lung [1,37–40]. To date, pendelluft is reported to be associated with longer ventilation duration in patients with severe ARDS [41], whereas its effect on patients with mild ARDS is unclear.

Despite several studies having focused on the harmful effects of pendelluft, its leading causative factors are not fully understood. To investigate these factors, a computer simulation study is suitable because it can handle a three-dimensional model and visualize the airflow inside the tracheobronchial tree under arbitrary boundary conditions imitating various respiratory status, such as the intensity of mechanical support and inspiratory effort [25–30]...”. (Page 18 line 345 to 356 of the revised text)

**Comment 2***. Method*

*interesting and challenging. You use of finite element method to simulate regional conditions along bifurcations of the tracheobronchial tree. But there are certain methodological concerns that need to be clarified.*

*2) In “investigating the effect of an ETT on pendelluft”, I do not understand the rationale for using non-invasive models.*

**Response**: We appreciated the advice to facilitate the study.

We used non-invasive models because we wanted to focus on the resistance of the ETT, by comparing the two models. The resistance of the ETT is reportedly high because of its small cross-sectional area. During the inspiratory phase, part of the inspiratory pressure generated by the ventilator and the patient’s diaphragm is consumed by the airway resistance, and the remaining pressure is used for lung inflation. Therefore, if the same inspiratory pressure were applied, a higher resistance of ETT could cause less ventilatory support. A previous report indicated that higher “external resistance” caused by ETT worsens the pendelluft in spontaneous breathing subjects.

Therefore, to emphasize the ETT resistance, we have added some sentences and references in the Introduction section as follows:

“Contrarily, ETT resistance is reported as the leading cause of lung edema [15,16] and pendelluft [13] under spontaneous breathing conditions. The pressure dissipation by the ETT affects the pressure delivered to the patient’s airway and gas flow in the patient’s lung because the pressure delivered to the airways is a residue of ventilator-generated pressure minus pressure dissipation caused by the ETT [17–20].”.

(Page 4 line 72 to 76 of the revised text)

Moreover, ETT affects the pressure and velocity of the patient central airway. Therefore, we hypothesized that the distribution of the gas flow toward each lung lobe might also be affected by ETT. Thus, to explain why we use these two models, we have added the following text in the Introduction section:

“By comparing these two models, we can examine the ETT’s role as an external resistance and jet flow distributer, and how the ventilatory support affects it.” (Page 4 line 88 to 90 of the revised text)

**Comment 3.** *These model omits that the vigorous contraction of the diaphragm is relevant to regional strain generation in basal regions, resulting in an imperfect elastic anisotropic inflation and amplifying the regional lung injury (and regional progression of pulmonary edema, tidal recruitment, etc).*

**Response:** We greatly appreciate the reviewer’s valuable suggestion. Understanding the concept of regional strain is crucial in comprehending the mechanisms of lung damage. As emphasized by the reviewer, our model does not directly describe the regional strain generation. The strain is defined as the ratio between the ventilatory volume (Δvolume) and a reference volume. Uneven flow distribution is associated with uneven Δvolume, meaning that greater flow towards lower lobes, as observed in this study, indicates the generation of regional strain in those areas.

Regrettably, we were unable to obtain the necessary compliance data for each lung lobe, which can vary during inspiration, particularly under ARDS conditions. Consequently, we were unable to replicate the entire inspiratory simulation. Instead, our focus was directed towards the early inspiratory phase, wherein we identified the uneven flow rate directed towards each lung lobe. This observation potentially reflects the occurrence of regional strain resulting from solid-like behavior.

To address this limitation, a previous study suggested employing a combination of lung CT images and the finite element method, which holds promise for resolving this issue. More comprehensive studies are needed, wherein the regional strain of each lung portion is accurately calculated, leading to a better understanding of the mechanisms underlying lung damage.

So, we added some sentences and references in the limitation section as follows:

“More clinical data, including data about the compliance of each lung lobe throughout the respiratory phases, will help us clarify the non-uniform inflation of the lung and regional strain [66].” (Page 22 line 454 to 456 of the revised text)

Additionally, regarding the regional progression of pulmonary edema, we added some sentences and references in the Introduction section as follows:

“Contrarily, ETT resistance is reported as the leading cause of lung edema [15,16] and pendelluft [13] under spontaneous breathing conditions.” (Page 4 line 72 to 73 of the revised text)

**Comment 4**. *It also does not consider the use of PEEP, inspiratory time, flow morphology or the role of chest wall compliance.*

**Response:** We agree with the reviewer’s comment. In this model, PEEP is not included in the simulation setting. Instead, the inlet pressure represents the ventilator’s driving pressure from the baseline (=PEEP), and the outlet pressure represents the patient inspiratory effort from the baseline, because this simulation is conducted under non-compression and steady state. As the reviewer pointed out, in clinical setting, PEEP affect the lung compliance, strain distribution pattern, and negative pressure generated by the diaphragm. However, there are limited available data on the relationship between the PEEP and inhomogeneous pressure distribution in the lungs by solid-like behavior.

So, we added some sentences and references in the limitation section as follows:

“Furthermore, the pressure and gas flow are affected by many respiratory factors. For example, PEEP affects the lung compliance, strain distribution pattern [62], and intensity of the spontaneous breathing [63,64].”. (Page 22 line 448 to 451 of the revised text)

Additionally, as the reviewer pointed out, inspiratory time, flow morphology, or chest wall compliance are important factors to determine the appropriate outlet boundary conditions. In this study, we could not simulate each of the factors separately because of the limited available data. Hence, the outlet boundary condition represents the Integrated value of these factors.

Although the precise value remains uncertain, assuming a higher chest wall compliance would necessitate the application of weaker negative pressure for outlet boundary conditions. This is due to the fact that a substantial portion of the patient’s inspiratory effort, which is in the form of negative pressure, is allocated towards chest wall movement rather than lung inflation.

As inspiration proceeds, pressure of the regional lung lobe (= outlet boundary condition) changes depending on these factors. In this study, we simulate “strong”, “moderate”, and “weak” outlet boundary conditions. “Strong” outlet boundary condition represents more negative pressure than “weak”. Hence, as inspiration proceed, flow condition is assumed to changes from “strong” to “weak”.

Thus, we added some sentences and references in the limitation section as follows:

“Because it is difficult to accurately recreate the boundary conditions across the entire respiratory cycle, we examined three intensities of inspiratory effort in this study. In practical terms inspiration proceeds and the lungs are inflated, the outlet boundary condition changes (corresponding to the intensity changes from strong to weak). Furthermore, the pressure and gas flow are affected by many respiratory factors. For example, PEEP affects the lung compliance, strain distribution pattern [63], and intensity of the spontaneous breathing [64,65]. Moreover, when higher chest wall compliance is assumed, a greater part of the patient’s inspiratory effort is used to move the chest wall than for lung inflation. As the boundary conditions used in this study are integrated values of these factors, the influence of each factor is not considered separately. More clinical data, including data about the compliance of each lung lobe throughout the respiratory phases, will help us clarify the non-uniform inflation of the lung and regional strain [66].” (Page 22 line 445 to 456 of the revised text)

**Comment 5**. *As I understand it, it models resistive component (purely frictional) and a sum of elastic and viscoelastic component of the lung parenchyma (as a whole, without separating both), omitting threshold component*.

**Response:** We agree with the reviewer’s useful comment.

As the reviewer pointed out, in this model, the resistive component is associated with the pressure difference between the inlet and outlet pressure, and a sum of elastic and viscoelastic component is associated with the outlet boundary conditions. The threshold component is the amount of work required to commence inspiratory flow and it is determined by auto PEEP. The threshold component is an important concept because it is associated with the work of the diaphragm to generate negative pressure. Regarding patients with ARDS, if the threshold component is high and thereby initiation of the inspiratory flow from the ventilator delayed, more gas movement from non-dependent to dependent lung can occur. Considering the result of this study that low ventilatory support deteriorate the pendelluft than high support, the inspiratory flow delay caused by threshold component (during this delay, the ventilatory support is zero) could deteriorate the pendelluft.

So, we added some sentences and references in the Discussion section (Comparative research 2 part) as follows:

“Regarding WOB, we should also consider the threshold load, which refers to the amount of work required to commence inspiratory flow; this is determined by auto-PEEP [61]. The inspiratory flow delay caused by the threshold load could worsen the pendelluft, because during this delay, the ventilatory support is zero, and more gas flow is assumed to be directed from the upper to the lower lobe.”

(Page 21 line 434 to 438 of the revised text)

**Comment 6**. *It assumes that the flow will be lower in all the regions studied in invasive conditions than in non-invasive conditions, which in a clinical setting will depend on the ventilatory drive and the capacity to generate force by the respiratory muscles.*

**Response:** We agree with the reviewer’s opinion.

Because of the narrow cross-section area of the endotracheal tube, the pressure decay between the inlet and outlet are larger in invasive model than non-invasive model. As the reviewer pointed out, the inspiratory flow depends on the ventilatory drive and the capacity to generate force by the respiratory muscles. Therefore, in the clinical setting, invasive conditions with appropriate ventilatory support have proved to be lung protective. In other words, invasive condition without appropriate ventilatory support can be harmful. As previously reported, the pressure decay caused by the ETT accounts for a certain percentage of total airway resistance. It is helpful to recognize ETT as external resistance, which cause excessive negative pressure and cause lung edema with spontaneous inspiratory effort.

So, we added some sentences and references in the Discussion section as follows:

“As shown in Table 1B, the gas flow rates are highly dependent on the inlet pressure. This means higher ventilatory support can compensate for the resistance of the ETT. Till date, appropriate ventilatory support has been shown to have a lung protective effect [1,2,36,44]. Therefore, we should recognize ETT as an external resistance [45–49] and should identify the resistive component from the total respiratory work [50–55].”

(Page 19 line 383 to 387 of the revised text)

**Comment 7**. *The authors should better explain how they defined (and measured) Pendelluft in their model.*

**Response:** We appreciate the reviewer’s comment.

In this study, as shown in table 1, the gas flow into the model (from inlet or outlet) are expressed as positive values, and gas flow out from the model are expressed as negative value. Intuitively, as this study simulates the inspiratory phase, the positive values are observed at inlet, and negative value are observed at outlet. Hence, we defined the pendelluft flow as positive value observed at outlet.

So, we added the following sentences in the Methods section:

“Under the boundary conditions, the gas flow out of the model through the outlets meant lung lobe inflation, whereas gas flow into the model through the outlets meant lung lobe deflation. Therefore, in this study, gas flow into the model through the outlets was defined as the pendelluft.” (Page 8 line 162 to 165 of the revised text)

We also added the following sentences in the Results section:

“The gas flow into the models was described as positive, and the gas flow out of the models was described as negative. Therefore, the positive value observed at outlets is defined as pendelluft.”

(Page 13 line 244 to 246 of the revised text)

**Comment 8**. Discussion

*Pendelluft refers to regional displacement or redistribution of gas during the inspiratory phase. While it is a potential stress raiser in severe ARDS, I am not sure it is always harmful.*

**Response**: We agree with the reviewer’s comment. As the reviewer pointed out, pendelluft is reported to be associated with longer ventilation duration in patients with PaO2/FiO2 ratio below 200 mmHg. However, the direct mechanism that pendelluft damage the lung is unclear. Theoretically, pendelluft is associated to the less homogeneous distribution of tidal volume, and the lung inhomogeneities may act regionally as stress raisers by multiplying the applied pressure. Then, the regional strain may cause the lung injury.

Therefore, we added some sentences and references to the Discussion section as follows:

“Recently, pendelluft has been considered one of the mechanisms of P-SILI, a concept wherein patients’ inspiratory effort cause regional edema, tidal recruitment, and distortion [1,2,35,36]. The inhomogeneous gas distribution of pendelluft is assumed to increase the strain and amplify the stress at the interface between open and closed pulmonary units (= stress raiser), thereby damage the lung [1,37–40]. to date, pendelluft is reported to be associated with longer ventilation duration in patients with severe ARDS [41], whereas its effect on patients with mild ARDS is unclear.” (Page 18 line 345 to 351 of the revised text)

**Comment 9**. Part of the discussion and abstract should be redirected according to observations made.

**Response**: We appreciate the reviewer’s comment. We rewrote the discussion and abstract to explain the relationships between lung damage, including P-SILI, and pendelluft, and discussed based on the observations.

In abstract, we added the following sentence:

“The airflow caused by this pressure difference is called pendelluft, which is a possible mechanisms of patient self-inflicted lung injury.”

In the Discussion section, we described how the observation in this study relates to previous findings on lung damage, including P-SILI, mechanical power and work of breathing. We also described the limitations of this study in more detail to explain the clinical implications of this research.
